# Confirmatory Validation of an Evaluation Instrument for Interventions Based on the Healthy Habits App Healthy Jeart with Adolescents

**DOI:** 10.3390/jpm12030470

**Published:** 2022-03-15

**Authors:** María Ángeles Merino-Godoy, Carmen Yot-Domínguez, Jesús Conde-Jiménez, Ana María de la Calle-Cabrera

**Affiliations:** 1 Department of Nursing, University of Huelva, 21071 Huelva, Spain; angeles.merino@denf.uhu.es; 2 Department of Didactics and Educational Organization, University of Seville, 41013 Seville, Spain; 3 Department of Theory and History of Education and Social Pedagogy, University of Seville, 41013 Seville, Spain; jconde6@us.es; 4 Department of Didactics of Language and Literature and Integrated Philologies, University of Seville, 41013 Seville, Spain; anamariadelacalle@us.es

**Keywords:** adolescents, eHealth app, health interventions, health promotion, instrument development

## Abstract

Mobile devices are widely used among young people, and their use for health promotion is in-creasing. Healthy Jeart is a mobile application aimed at promoting healthy life habits among people aged 8–16 years. The aim of this study was to develop and validate an instrument that allows evaluating the healthy knowledge, habits and attitudes learned by adolescents aged 12–16 years through the Healthy Jeart application. Attending to the content of Healthy Jeart, a first version of the evaluation instrument was generated. It was subjected to expert judgement. The second version was administered to 429 adolescents from six educational centres of Andalusia to carry out the validation of the construct through exploratory and confirmatory factor analyses. After exploration, a six-factor model was confirmed, with a very adequate level of fit and good internal consistency. The six factors were: (1) knowledge about eating and physical activity, (2) habits about eating and physical activity, (3) emotional health, (4) consumption of alcohol and drugs, (5) social relationships and (6) sexual activities and use of technologies. There are at least four instruments that could be used to measure health-promoting behaviours. However, this new instrument was created ad hoc. It measures exactly the results that can be expected. Healthy Jeart will now have a valid and reliable evaluation instrument: Ev-HealthyJRT v.1.0. Young people, teachers and other professionals who carry out health-promotion interventions based on Healthy Jeart with adolescents will have at their disposal an instrument integrated in this app that allows verifying the learning results. However, the validated instrument can be used for evaluation in other interventions, as long as the multiple and essential aspects of a healthy living are addressed as in Healthy Jeart.

## 1. Introduction

Healthy Jeart is a mobile application aimed at promoting healthy life habits among people aged 8–16 years. It is available, free of charge, for Android and iOS devices. It has been considered as a “healthy app” by the Andalusian Agency of Health Quality. This distinction is the first in Spain to recognise the quality and safety of health applications. Continuous work is carried out on Healthy Jeart to keep it up to date and functioning properly. This study presents the creation and validation of an instrument that allows evaluating the learning results about health in adolescents aged 12–16 years. This population group works in it on seven areas of health, of which two are restricted for children aged 8–11 years. The mentioned instrument will be implemented in the application to facilitate the verification of knowledge, attitudes and habits before, during and after the use of Healthy Jeart. Further information about the project can be found at http://www.healthyjeart.com/ (accessed on 14 March 2022).

## 2. Background

Adolescence is an ideal stage to promote a healthy lifestyle and correct unhealthy habits [1]. In general, adolescents do not have healthy lifestyles [2]. Moreover, during the transition to young adulthood (13–24 years), their healthy behaviours decline, giving rise to significantly less healthy practices [3]. Therefore, it is urgent to help them to improve their habits and sensitise them about the importance of avoiding risks to their present and future health state [4]. Adolescents should know that the early adoption of healthy habits strongly contributes to a better future quality of life [5].

The progressive development of educational interventions aimed at increasing the health of young people, in the school context and in other scopes, has generated the need to create valid instruments for the evaluation of health-promoting behaviours. Evaluation is an essential element of the design and development of interventions to promote lifestyles among adolescents. Therefore, the creation of valid instruments is an important challenge. Thus, there are different instruments related to healthy lifestyles in adolescents, such as those of Gillis [6] (ALQ, Adolescent Lifestyle Questionnaire), Chen et al. [7] (Adolescent Health Promotion Scale, AHPS), Hendricks et al. [8] (ALP, Adolescent Lifestyle Profile) and Mahon et al. [9,10] (revised PLQ, Personal Lifestyle Questionnaire for adolescents). The Adolescent Lifestyle Questionnaire is a 43-item instrument designed to measure healthy lifestyle practices in adolescents. It focuses on identity awareness (nine items), nutrition (eight items), physical participation (four items), safety (seven items), health awareness (four items), social support (seven items) and stress management (four items). The Adolescent Health Promotion Scale has 40 items and about six domains of healthy lifestyles (social support, life appreciation, health responsibility, nutritional behaviours, exercise behaviours and stress management). The Adolescent Lifestyle Profile scale initially had 42 items, and two more items were eventually added. The resulting new scale, Adolescent Lifestyle Profile Revised 2 (ALP-R2), has 44 items and seven dimensions (health responsibility, physical activity, nutrition, positive life perspective, interpersonal relationships, stress management and spiritual health). The Personal Lifestyle Questionnaire (PLQ) was developed by Muhlenkamp and Brown [11] to measure positive health practices in adults and older adolescents. Its version for early adolescents is a 24-item scale that is internally organised in two factors (Health Promotion Practices and Health Protective Practices) and six subscales (nutrition, four items; relaxation, five items; exercise, four items; health promotion, four items; safety, four items; and substance use, three items). These have been revised, adapted and validated transculturally [12,13,14,15]. In addition to these instruments, others such as ENHASA [16] are considered effective questionnaires. This questionnaire assesses the main modifiable health behaviours among adolescents aged 12–14 years related to eating (eight items), physical activity (four items), new technologies (seven items), and environment (seven items). Its use is indicated for schools, due to the specificity of some of its items, such as “brings lunch from home”.

Currently, health interventions are being mediated by technology [17]. Mobile devices are widely used among young people, and their use for health promotion is increasing. Thus, in the scientific literature, we find documented interventions with eHealth apps, such as that of Benavides et al. [18]. Specifically, eHealth interventions that address multiple lifestyle risk behaviours have been confirmed as effective at improving physical activity and eating, although their effects are small and evident only immediately after the intervention [19]. The research studies on health promotion with applications are still limited; however, the use of applications seems to be viable and promising [20]. Fedele et al. [21] concluded that mobile health interventions have a significant effect, even though small, on the health of young people. They improve several lifestyle domains, such as physical activity [22], eating, health responsibility and positive life perspective [23].

### Healthy Jeart

Healthy Jeart is a free health application for children and adolescents aged 8–16 years. Its first version was published in 2018. Before that, the Andalusian Agency of Health Quality (ACSA) evaluated it as a “healthy app” [24]. This app was designed by researchers of the Universities of Seville and Huelva [25] to promote healthy lifestyles with a global approach on health. It fits the needs and concerns of young people, as the design process was initiated with the development of nominal groups with which relevant topics to address were identified [26].

**Figure 1:**
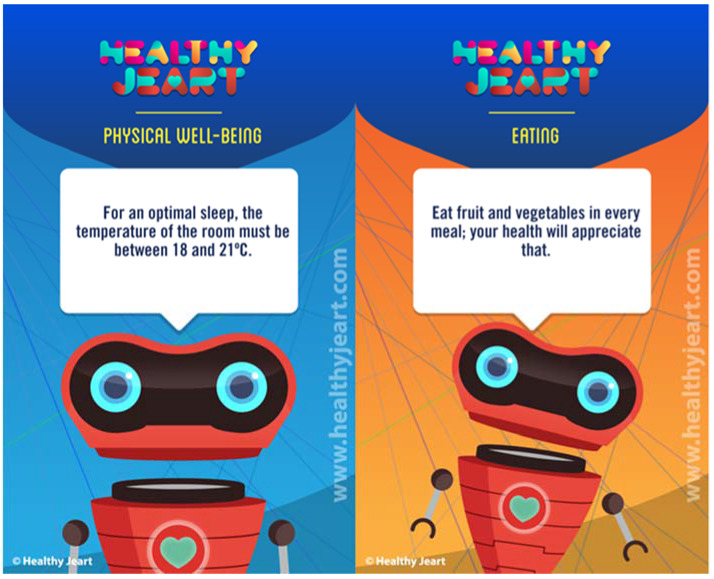
Examples of app tips.

Minors can find in it clear, brief, simple and understandable messages (called tips), with practical advice and suggestions, with a theoretical basis, on different areas of health. Young people aged 12–16 years have information about the following seven areas: physical activity (physical state, physical exercise, sport), eating (nutrition, food and drinks), physical well-being (sleep, personal hygiene and safety, time management), psychological well-being (self-esteem and self-concept, emotions, social skills), toxic substances and addictions (substances such as alcohol, tobacco, cannabis), affection-sex (sexuality, couple relationships, gender violence) and new technologies (use and abuse of technologies, social networks). Children aged 8–11 years do not have access to the areas on toxic substances and addiction and affection-sex. The aim is to help them expand their knowledge in every area, develop favourable attitudes and become aware of how they can improve their habits and adopt healthy behaviours. Experts in the corresponding areas have approved the tips of each of the areas. All of them have been written for children and young people, using strategies to help them remember, such as rhymes and jokes (in Figure 1 two examples of tips from the app are shown).

In addition to the tips, an attractive game helps them to recognise the different basic and important aspects of health. Jeart, a heart-shaped friendly alien, is the main character. Jeart must keep moving by making a vertical tour. As it goes up, Jeart must come into contact with objects that represent aspects of the different areas that favour health (e.g., sports, water, sleep) and avoid products and activities that are bad for health (e.g., drugs, sugar, soft drinks). In a forum where healthy ideas can be shared, the users can obtain rewards for the game.

Healthy Jeart is designed for use in the school context, although it can also be used in other areas. Therefore, this application provides teachers with proposals of educational activities for each of the areas. The work on the content addressed in the app extends to the classroom under the guidance and counseling of the teacher through applied specific activities, which can be performed individually or in small or large groups. Moreover, Healthy Jeart also provides monthly health challenges in which groups-classes can participate and compete with those of other educational centres. These are propositions of activities that must be conducted in a continuous manner, with the aim of consolidating habits such as relaxation and hydration, among others.

The research team involved in the design and production of the application have worked in the writing and validation of an evaluation instrument that allows measuring the knowledge, habits and attitudes about health that are addressed in the app for young people aged 12–16 years. It is necessary to accompany the content and work propositions with an instrument that accurately measures the results, specifically considering those that can be achieved. Resorting to non-specific instruments would distort the initial, continuous or final evaluation in interventions with Healthy Jeart. The few instruments available to date do not fully fit our evaluation purpose. The Adolescent Lifestyle Questionnaire [6] includes a domain of stress management, which is not directly addressed in Healthy Jeart. Its safety domain considers the rejection of the consumption of substances that are harmful to health and safety in sexual relations, although, on the latter, there are only two items, which is insufficient to cover the affection-sex area of Healthy Jeart. In addition, this instrument does not expressly refer to the responsible and safe use of mobile devices and digital social networks. Similarly, the Adolescent Lifestyle Profile Revised 2 [8,12,14] and the Adolescent Health Promotion Scale [7] do not cover the new technologies area of Healthy Jeart. If those instruments were used, the areas of toxic substances and addictions and affection-sex would not be evaluated. In ALP-R2, there are items that do not correspond to the content of the other areas, such as “I seek guidance from school counselor when needed” and “I attend a group that shares my spiritual beliefs”. Spiritual health is not an area of Healthy Jeart. The revised Personal Lifestyle Questionnaire for adolescents [9] does not address areas expressly worked in the app, such as affection-sex and new technology. With it, very specific behaviours that are not promoted in the app would be valued as “limit caffeine intake to three cups daily (includes sodas, coffee, and tea)”. The purpose is to create a self-report instrument that addresses the current health areas included in the app, adjusts to the real needs and interests of the adolescents and can be used in the context of mobile interventions, without being limited to the context of the school.

## 3. Method and Materials

The general aim of this study was to design and validate an instrument that allows evaluating the healthy knowledge, habits and attitudes learned by adolescents aged 12–16 years through Healthy Jeart.

This is a quantitative, cross-sectional study. The creation of the instrument involved work that was clearly differentiated into two phases. First, we wrote the items and validated the content of the instrument. Second, we conducted the validation of an explanatory model that supports the instrument.

Primary identification of the items to incorporate in the new instrument was accomplished using a complete review of the app. Attending to the content of Healthy Jeart, the first version of the evaluation instrument was generated. As was already mentioned, this content has theoretical foundation and was validated when the app was designed and produced by experts. It was also evaluated by the ACSA. From thorough analysis of the tips, activities and challenges available in the application, 72 items were extracted. These items correspond to the different areas of the app in the following manner: 1. Physical activity (nine items); 2. Eating (eleven items); 3. Physical well-being (nine items); 4. Psychological well-being (fifteen items); 5. Toxic substances and addictions (seven items); 6. Affection-sex (eleven items), and 7. New technologies (ten items). These are some of the items: “I avoid doing sedentary activities like watching TV or play videogames in my spare time”; “I have personal hygiene habits like brushing my teeth after each meal” and “I know what to do in situations of gender violence”. The revision of the standardised instruments available in the scientific literature inspired the wording of the items. Thus, similarities can be found with these.

This instrument was subjected to expert judgement for content validation. A total of 15 experts (eight women and seven men; mean age: 46 years) were asked through a digital questionnaire. These experts worked in the Andalusian Healthcare Service (four), local administration (three), educational centres and universities (six) and non-for-profit organisations (two). The expert group valued from 1 (Totally disagree) to 10 (Totally agree) the following criteria per item: clarity in the writing (the item is clearly written); internal coherence (the writing of the item is coherent); response induction (the writing of the item induces the answer); suitability of the language with the level of the respondents (the writing of the item suits the age range of 12–16 years), and target evaluation (the item evaluates exactly what it expresses). We considered that a mean score equal to or higher than 8 points per item and criterion showed an adequate degree of agreement. All the items surpassed this mean score in all the different criteria.

Moreover, the experts expressed their perception toward the instrument in its entirety through the following five items: 1. The instrument contains clear and precise instructions to be answered; 2. The items allow evaluating the content of the Healthy Jeart app; 3. The items are distributed in a logical manner; 4. The items are organised correctly, and 5. The items are free of spelling errors. The degree of agreement with each of the items was expressed in a scale of 1 (Totally disagree) to 10 (Totally agree). Table 1 presents the means obtained for each item, which were all above nine points. The standard deviation was below 1, indicating little variation.

However, along with the valuations, the experts made qualitative observations for each item and for the instrument in its entirety. Some of such observations suggested minor changes in the writing of the items to better suit the age of the end recipients, such as: “I would replace I opt with I choose”; “I would also add walking as an example”; “Indicate the main foods of the Mediterranean diet”, and “I would replace continuity with perform regularly”. Other comments encouraged the formulation of new items, e.g., “These are two items in one. I can differentiate the groups of foods, but not the amount, and that could make the users doubt”. Consequently, the writing of all the items was revised attending to the suggestions and three new items were included in the instrument. In the eating area, the item “I differentiate the groups of foods and the amount of them that we must consume daily, according to the food pyramid” was fragmented into the following two items “I differentiate the groups of foods of the food pyramid,” and “I know the amount of each food group that we should consume daily according to the food pyramid”. In this area, the following item was included, to satisfy the request of most of the experts: “I search for information to improve my nutrition”. In the physical well-being area, the item “I have healthy routines such as sleeping at least 8 h and drinking at least 2 litres of water every day” was replaced with the following two items: “I try to sleep at least 8 h every day” and “I drink at least 2 litres of water every day”.

Thus, the second version of the instrument had 75 items, which represented each of the areas in the following manner:

1. Physical activity (nine items): the extent to which young people know the importance of practising physical activity and do so safely.
2. Eating (thirteen items): the knowledge that young people have about nutrition (food pyramid, saturated and unsaturated fats, ultra-processed foods, etc.) and the habits they must have to keep a healthy diet.
3. Physical well-being (ten items): the habits of hygiene, personal care and safety that young people have acquired to protect their own body.
4. Psychological well-being (fifteen items): the extent to which young people recognise their emotions and reflect on their needs, have a positive attitude toward problems and conflicts and nurture their relationships with others.
5. Toxic substances and addictions (seven items): the knowledge that young people have about alcohol and drugs and their attitude toward their consumption.
6. Affection-sex (eleven items): the extent to which young people recognise the link between sexuality and identity, their capacity to differentiate between love, attraction and infatuation, and what they know about how having consented and safe sexual relations.
7. New technologies (ten items): the use that young people make of mobile devices, how they behave in social networks, their capacity to recognise that the excessive use of mobile phones generates dependency, and that social networks can be dangerous.

Next, after obtaining the permission to access the centres from the school principals and the participation consent authorised by the families, the instrument was administered to a sample of young people from six educational centres of the provinces of Huelva and Seville (Andalusia, Spain). The 75 items were presented in a five-point Likert scale of 1 (Totally disagree) to 5 (Totally agree), after a series of demographic questions, such as sex, course, educational centre and locality.

The second version of the instrument and the document of informed consent of participation for the families were printed and sent to the principals of the participating educational centres. They distributed them among the tutor teachers in each group of students. These teachers were asked to inform the respective families and collect the signed consent forms, as well as to subsequently explain the purpose of the study and administer the instrument to the students in class. The students responded to the instrument in paper format during the tutoring hour assigned in the weekly timetable. In the classroom, one of the researchers of the team was present to offer support. Previously, they had to have interacted with the app at home. The data gathering process took place in April of the academic year 2020/2021. The responses were loaded to a database of the statistical software IBM SPSS Statistics version 26.0 in Spanish (Madrid, Spain).

### 3.1. Ethical Considerations

This study strengthens the functionality of Healthy Jeart, with the creation and validation of Ev-HealthyJRT v.1.0, an evaluation instrument that will be implemented in it. For the validation of the evaluation instrument, it was necessary to collect self-reported data from the adolescents. To this end, authorisation from their families was obtained through a document of informed consent for participation. The anonymity of the answers was guaranteed at all times. No videos, images or audio recordings were taken during the filling of the questionnaire. The purpose of participation was to prove the validity of the instrument (assess its psychometric properties), and the teachers, students and families were informed about it. Students participated in the study freely.

This study is framed within the research project “Design of a mobile phone application to educate the youth in healthy habits”. The Research Ethics Committee of the province of Huelva approved the project. The secretary of its Standing Committee recently issued a certificate dated 19 November 2021.

### 3.2. Sample/Participants

The participants were recruited by non-probabilistic, convenience sampling. The inclusion criteria were: (1) age between 12 and 16 years; (2) enrolment in an educational centre of Huelva or Seville with authorisation of the principal to participate in the study and access to the centre; (3) informed consent of the parents or guardian prior to participating in the study, and (4) previous knowledge and usage of the app. The guaranteed sample size was of 300 students, i.e., the minimum required to perform a factor analysis.

The sample of participants was constituted by 429 Secondary Education students from six educational centres of the Andalusian provinces of Huelva and Seville, of which three were public centres and the other three were private-charter centres.

Regarding gender, the sample was equally distributed (51% boys and 49% girls). The age of the participants ranged between 12 and 16 years, with an average age of 14 years (SD: 1.156). Most of the students (89.5%) were between 12 and 15 years old. Of the four courses that integrate Secondary Education in Spain, 25.4% of the participants belonged to Year 8, 31.9% to Year 9, 30.8% to Year 10 and 11.9% to Year 11.

### 3.3. Data Analysis

The data were subjected to a factor analysis (first exploratory and then confirmatory analysis), using JASP open source software version 0.11.1.0 in Spanish (Department of Psychological Methods, University of Amsterdam, Amsterdam, The Netherlands), to define an explanatory model about the study object. IBM SPSS Statistics software v.26 (Madrid, Spain) was used for the descriptive statistical analyses of the mean, variance, standard deviation, range, minimum and maximum values.

At the procedural level, firstly, an exploratory factor analysis (EFA) was conducted. To estimate the number of factors, a parallel analysis was applied following the minimum residual estimation method and promax oblique rotation, obtaining a first approximation of the items to be included in the model. This first analysis helped to propose a reference structure. This analysis revealed a seven-factor model. However, the exclusion of items with a low factorial (below 0.4) resulted in readjustment to an explanatory model with six factors. Thus, this analysis revealed six factors for the organization of 32 items, with adequate fit indices (RMSEA = 0.037 and TLI = 0.83). After this exploration, those elements that did not have a factorial load between 0.4 and 1 were eliminated again, which reduced the initial theoretical design proposal from 75 items to 31. The confirmatory analysis work continued with these 31 items.

Secondly, a confirmatory factor analysis (CFA) was performed. To determine the goodness of fit of this model, we analysed additional fit parameters, the goodness of fit index (GFI), and the coefficient of determination (R^2^). We took as reference the criterion established by Hair et al. [27] and Hoyle [28], according to which, good values of fit are obtained if GFI and CFI ≥ 0.96, TLI ≥ 0.95, and RMSEA ≤ 0.05; moderate values if CFI, GFI and TLI ≥ 0.90 and RMSEA ≤ 0.08; and low values if CFI, GFI and TLI ≤ 0.90 and RMSEA ≤ 0.10. Regarding the Standardized Root Mean Square Residual (SRMR), we applied the criterion of Hair et al. [27] and Hu & Bentler [29], which consider that values equal to or lower than 0.08 indicate a good fit. Different tests were carried out for the exclusion of items and their reorganization in the factors, seeking the best possible fit of the model, taking into account the factor loadings and the theoretical dimensions (areas) worked on in the app. For this reason, the confirmed model included items with factor loadings greater than or around 0.4, since this contributed to the improvement of its fit indexes.

## 4. Results

The resulting model derives from the solution obtained in the CFA based on it to the first examination (EFA), undergoing adjustment in the items included (based on their factorial load) and their reorganization within the factors (based on the areas), as indicated in the previous section on data analysis. Thus, the model derived from this first exploration was tested, which is made up of six factors. Of the 31 items that were integrated into the six factors, 24 of these were included in the confirmed model, since a satisfactory adjustment solution was obtained.

Likewise, a unidimensional model was estimated that presented worse fit indices χ^2^ (252, *n* = 429) = 1331.94, *p* < 0.001; RMSEA = 0.100 (95% CI [.095, 0.105], CFI = 0.61, NNFI = 0.57, SRMR = 0.09. Consequently, based on the results obtained, the model of six correlated factors was chosen, made up of 24 items, showing better fit indices as detailed below.

Factor 1 is knowledge about eating and physical activity. It includes four items from the physical activity and eating areas. Factor 2, habits about eating and physical activity, has three items also from these two areas. Emotional health is the third factor. Its four items come from the psychological well-being area. Consumption of alcohol and drugs (Factor 4) and social relationships and sexual activities (Factor 5) have four and five items, respectively. These factors fully correspond to the toxic substances and addictions and affection-sex areas in the same way as Factor 6 (use of technologies), with three items, and are associated with the new technologies area.

First, the confirmed model presents a Chi-squared distribution with values of χ^2^ = 470.013 and *p*-value < 0.001, as is shown in Table 2.

The Comparative Fit Index (CFI) and Tucker-Lewis Index (TLI) of the model, as presented in Table 3, obtained the following values: CFI = 0.916 and TLI = 0.902. Thus, the explanatory model obtained moderate parameters of fit in these indices.

The Root Mean Square Error of Approximation (RMSEA) (in Table 4) obtained a value of 0.046 (95% CI: [0.040, 0.051]). Moreover, the confidence intervals are above 0, do not change sign and are under 0.06, indicating that the hypotheses of the model are not due to chance. Table 4 also shows the values of Goodness of fit index (GFI), which was 0.914, and Standardised Root Mean Square Residual (SRMR), which was 0.061. Both indicate good results of goodness of fit.

Regarding the factor loading of the items of the confirmed model, these are around or above 0.4 (see Table 5). All these items were incorporated, due to the adequate indices and parameters of fit obtained, as was previously shown. As is shown in Table 5, *p*-values under 0.001 were obtained. This reveals that each item is different from the others. Moreover, the confidence intervals are positive and there is no change of sign, thus value 0 does not apply, i.e., this hypothesis does not admit chance.

Table 6 shows the residual variances by item. *p*-values below 0.001 were obtained, as well as positive confidence intervals without change of sign.

**Figure 2:**
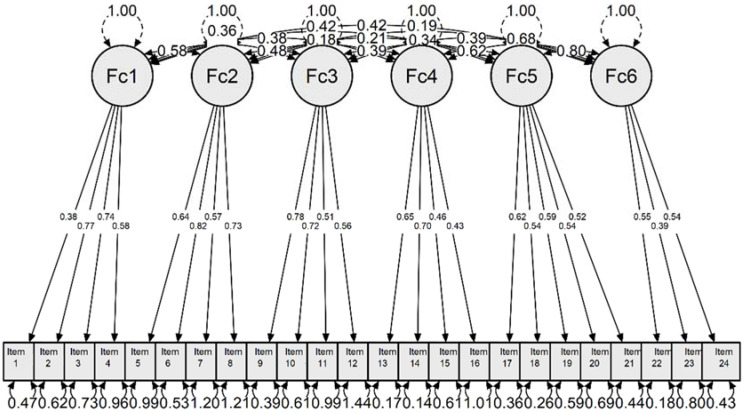
Graphic representation of the model.

Next, in Figure 2 the confirmed model is presented in a graphical manner.

As shown in Table 7, the correlations between the factors are significant, since *p* values are obtained below 0.005. The confidence intervals are positive and, most importantly, there is no sign change.

To determine the reliability of the instrument, we applied the criterion that a Cronbach’s alpha coefficient above 0.80 is considered adequate. In our case, we obtained a Cronbach’s alpha of 0.837, indicating a good internal consistency. Likewise, taking into account the McDonald’s omega coefficient, a good reliability of the model is obtained, since it is above 0.8. See Table 8.

Lastly, we present the mean values obtained in each item for each of the factors that integrate the resulting model (in Table 9). In general, high average scores were obtained. Regarding Factors 4 and 5, which were named “Social relationships and sexual activities” and “Consumption of alcohol and drugs”, respectively, all the items obtained a mean score above 4. In factor 6, named “Use of technologies”, the same positive tendency was observed. In Factor 1, named “Knowledge about nutrition and physical activity”, most of the items obtained mean scores between 3 and 4 points; only item 1 (“I know the benefits of practicing physical activity daily”) was above a mean score of 4 points. In Factor 3, named “Emotional health”, the score of the items was between 3.5 and 4, obtaining moderate mean values. The lowest mean scores were those of Factor 2, named “Habits related to eating and physical activity”, ranging between 2.6 and 3.6 points. In this sense, item 8 (“I search for information to improve my nutrition”) stands out, with a mean score of 2.59 points, which was the lowest score of the scale.

## 5. Discussion

Currently, humans are going through complicated years, in every scope of life, due to the disease associated with SARS-CoV-2 [30], which, shortly after its appearance, became an international concern as a public health emergency [31]. In Spain, the pandemic had an impact on all the elements of the healthcare system [32], which suffered a collapse similar to that observed all over the world. This meant a radical change in practising family and community nursing, since the promotion of education and health was affected because educational consultations for sick or healthy children and adolescents were inhibited or canceled.

The promotion of health at these ages is vital. Interventions with adolescents aimed at improving health are necessary, and technologies, including mobile applications, are optimal tools on which to base such interventions. Thus, the WHO [33] recently published a guide for their design, development and implementation. Healthy Jeart is an app for young people specifically created to promote healthy life habits. Its development and distribution were justified by the increasing use of eHealth apps, and now its usefulness is strengthened by the prevailing need for having tools that allow carrying out remote and blended interventions based on technology [34]. This year, we worked on expanding the functionality of Healthy Jeart. This study presents the process of creating and validating an instrument that allows evaluating, at any time, the knowledge, habits and attitudes of adolescents, specifically those seen in the app, with reliable results. To guarantee the validity of the work, we validated the content, through an expert panel, and the construct, through EFA and CFA. Moreover, to ensure cognitive validity, a researcher of the team was present throughout the entire data gathering process.

The confirmed model, represented in Ev-HealthyJRT v.1.0, consists of six factors. These correspond to the areas considered in Healthy Jeart for internal organisation in the following manner: knowledge about eating and physical activity and habits related to eating and physical activity (with Eating and Physical activity); emotional health (with psychological well-being); consumption of alcohol and drugs (with toxic substances and addictions); sexual relationships and sexual activities (with affection-sex) and use of technology (with new technologies). Moreover, it should be noted that the only area (theoretical dimension) of the app that is not constituted as a factor within the model is physical well-being. Therefore, the resulting instrument is reduced to the app essence and that area is the only one whose content could be diluted in the rest, specifically with the factors 1 and 2.

There are at least four instruments that could be used to measure health-promoting behaviours, including ALQ [6], AHPS [7], ALP [8] and PLQ [9,10]. Among these instruments, there are factors and items that coincide with each other, as well as with the instrument that we have developed and validated. However, this instrument was created ad hoc, that is, it is based on the content of the application itself and written in the same terms as the information. Consequently, it measures exactly the results that can be expected. This would not be possible with any of the previous instruments.

The generated instrument is highly specific. Its usefulness is mainly linked to the use of Healthy Jeart. Therefore, the instrument will be especially useful to its end users: adolescents, teachers and professionals with responsibility in the promotion of health. However, if we look at the wording of its items, and compare them with those of other instruments, we can see that the validated instrument can be extrapolated and used for evaluation in other interventions as long as they address multiple aspects of a healthy life as in Healthy Jeart. The main strength of the instrument with respect to the existing ones is that it is more current. The main risks that adolescents may assume regarding the consumption of toxic substances, sexual activities or technology usage must be considered in health promotion interventions. Thus, the knowledge, attitudes and behaviours to be promoted with respect to them are expressly measured with Ev-HealthyJRT v.1.0.

### Limitations

This study has some limitations that must be highlighted, such as the contact made with the educational centres. The protection and safety measures adopted due to the impact of COVID-19 forced us, firstly, to work only on the evaluation instrument for young people aged 12–16 years. In the academic year 2020/2021, it was very difficult to contact students through their centres. Moreover, we could not address the instrument for children aged 8–11 years. Secondly, we had to choose a convenient sampling method and resort to gathering data in those centres with associated nursing professionals known to the research team. In this respect, it is also worth highlighting that the sample size could have been larger and the population could have been better represented.

## 6. Conclusions

Young people aged 12–16 years, teachers and professionals who carry out health promotion interventions based on Healthy Jeart with adolescents, will have an integrated instrument that will allow verification of the results. We will continue to work on the design and validation of another instrument that may be used with children aged 8–11 years, as we believe that, when implementing an intervention, it is essential to measure the results coherently and clearly.

According to the indicators of fit obtained in the results, the instrument in its first version (v.1.0) has good psychometric properties to evaluate the knowledge, habits and attitudes of the adolescent population who use Healthy Jeart. Ev-HealthyJRT, in its first version (v.1.0), is a useful instrument. However, we propose the possibility of working on other versions of the instrument as the app is updated, to always adjust to the content, and as our understanding about risks in adolescence grows.

## 7. Patents

The necessary documentation to declare the authorship of the instrument presented here in digital format has been presented at the Office for the Transfer of Research Results (OTRI) of the University of Huelva (Huelva, Spain), as a patent. The specific name of the instrument is “Ev-HealthyJRT v.1.0. Escala de evaluación para adolescentes de conocimientos, hábitos y actitudes de salud, versión 1.0” and can be found at http://www.healthyjeart.com/ (accessed on 19 January 2022), created by Mª Ángeles Merino-Godoy (University of Huelva, Huelva, Spain), Carmen Yot-Domínguez (University of Seville, Seville, Spain), Jesús Conde- Jimenez (University of Seville, Seville, Spain) and Daniel Martin-Gil (University of Huelva, Huelva, Spain).

## Figures and Tables

**Table 1:** Descriptive statistics derived from the validation of the content through the expert judgement.

	The Instrument Contains Clear and Precise Instructions to Be Responded	The Items Allow Evaluating the Content of the Healthy Jeart App	The Items Are Distributed in a Logical Manner	The Items Are Organised Correctly	The Items Are Free of Spelling Errors
Mean	9.20	9.33	9.67	9.60	9.53
Standard Deviation	0.941	0.816	0.488	0.507	0.640
Minimum	7	8	9	9	8
Maximum	10	10	10	10	10

**Table 2:** Chi-squared distribution.

Model	χ²	df	*p*
Baseline model	3038.601	276	
Factor model	470.013	237	<0.001

**Table 3:** Fit indices.

Index	Value
Comparative Fit Index (CFI)	0.916
Tucker-Lewis Index (TLI)	0.902
Bentler-Bonett Non-normed Fit Index (NNFI)	0.902
Bentler-Bonett Normed Fit Index (NFI)	0.845
Parsimony Normed Fit Index (PNFI)	0.726
Bollen’s Relative Fit Index (RFI)	0.820
Bollen’s Incremental Fit Index (IFI)	0.917
Relative Noncentrality Index (RNI)	0.916

**Table 4:** Other fit measures.

Metric	Value
Root mean square error of approximation (RMSEA)	0.046
RMSEA 90% CI lower bound	0.040
RMSEA 90% CI upper bound	0.051
RMSEA *p*-value	0.892
Standardized root mean square residual (SRMR)	0.061
Hoelter’s critical N (α = 0.05)	260.180
Hoelter’s critical N (α = 0.01)	274.614
Goodness of fit index (GFI)	0.914
McDonald fit index (MFI)	0.745
Expected cross validation index (ECVI)	1.564

**Table 5:** Factor loadings.

							95% Confidence Interval	
Factor	Indicator	Symbol	Estimate	Std. Error	*z*-Value	*p*	Lower	Upper
Factor 1	Item 1	λ11	0.382	0.042	9.176	<0.001	0.300	0.463
	Item 2	λ12	0.768	0.056	13.706	<0.001	0.658	0.878
	Item 3	λ13	0.741	0.058	12.791	<0.001	0.627	0.855
	Item 4	λ14	0.577	0.060	9.648	<0.001	0.460	0.694
Factor 2	Item 5	λ21	0.635	0.062	10.201	<0.001	0.513	0.757
	Item 6	λ22	0.815	0.057	14.378	<0.001	0.704	0.927
	Item 7	λ23	0.567	0.066	8.598	<0.001	0.438	0.696
	Item 8	λ24	0.725	0.069	10.467	<0.001	0.589	0.861
Factor 3	Item 9	λ31	0.782	0.050	15.506	<0.001	0.684	0.881
	Item 10	λ32	0.718	0.054	13.375	<0.001	0.613	0.823
	Item 11	λ33	0.505	0.058	8.631	<0.001	0.390	0.620
	Item 12	λ34	0.559	0.070	7.997	<0.001	0.422	0.697
Factor 4	Item 13	λ41	0.652	0.033	20.037	<0.001	0.588	0.716
	Item 14	λ42	0.697	0.033	21.274	<0.001	0.632	0.761
	Item 15	λ43	0.463	0.043	10.687	<0.001	0.378	0.548
	Item 16	λ44	0.428	0.054	7.928	<0.001	0.322	0.533
Factor 5	Item 17	λ51	0.620	0.039	15.763	<0.001	0.543	0.697
	Item 18	λ52	0.536	0.034	15.986	<0.001	0.470	0.601
	Item 19	λ53	0.588	0.046	12.782	<0.001	0.498	0.678
	Item 20	λ54	0.538	0.048	11.141	<0.001	0.443	0.633
	Item 21	λ55	0.515	0.040	12.943	<0.001	0.437	0.593
Factor 6	Item 22	λ61	0.548	0.033	16.831	<0.001	0.484	0.611
	Item 23	λ62	0.394	0.050	7.842	<0.001	0.296	0.493
	Item 24	λ63	0.542	0.041	13.257	<0.001	0.462	0.622

**Table 6:** Residual variances.

					95% Confidence Interval	
Indicator	Estimate	Std. Error	z-Value	*p*	Lower	Upper
Item 1	0.472	0.036	13.059	<0.001	0.401	0.543
Item 2	0.624	0.065	9.666	<0.001	0.498	0.751
Item 3	0.735	0.069	10.691	<0.001	0.600	0.869
Item 4	0.957	0.074	12.852	<0.001	0.811	1.103
Item 5	0.990	0.080	12.430	<0.001	0.834	1.146
Item 6	0.534	0.067	7.943	<0.001	0.402	0.665
Item 7	1.196	0.091	13.212	<0.001	1.019	1.373
Item 8	1.207	0.098	12.268	<0.001	1.014	1.400
Item 9	0.390	0.055	7.158	<0.001	0.283	0.497
Item 10	0.610	0.059	10.304	<0.001	0.494	0.726
Item 11	0.986	0.073	13.449	<0.001	0.842	1.130
Item 12	1.440	0.106	13.645	<0.001	1.233	1.647
Item 13	0.172	0.020	8.474	<0.001	0.132	0.212
Item 14	0.140	0.021	6.660	<0.001	0.099	0.181
Item 15	0.607	0.044	13.913	<0.001	0.521	0.692
Item 16	1.009	0.071	14.275	<0.001	0.871	1.148
Item 17	0.362	0.032	11.432	<0.001	0.300	0.424
Item 18	0.259	0.023	11.283	<0.001	0.214	0.303
Item 19	0.588	0.046	12.887	<0.001	0.499	0.677
Item 20	0.694	0.052	13.403	<0.001	0.593	0.796
Item 21	0.437	0.034	12.828	<0.001	0.370	0.504
Item 22	0.177	0.022	7.894	<0.001	0.133	0.221
Item 23	0.800	0.057	13.963	<0.001	0.688	0.912
Item 24	0.428	0.035	12.126	<0.001	0.359	0.497

**Table 7:** Factor Covariances.

							95% Confidence Interval	
			Estimate	Std. Error	z-Value	*p*	Lower	Upper
Factor 1	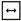	Factor 2	0.575	0.055	10.456	<0.001	0.468	0.683
Factor 1	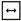	Factor 3	0.360	0.061	5.870	<0.001	0.240	0.480
Factor 1	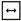	Factor 4	0.376	0.056	6.760	<0.001	0.267	0.485
Factor 1	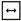	Factor 5	0.417	0.057	7.301	<0.001	0.305	0.529
Factor 1	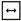	Factor 6	0.419	0.061	6.923	<0.001	0.300	0.538
Factor 2	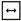	Factor 3	0.476	0.057	8.306	<0.001	0.364	0.588
Factor 2	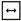	Factor 4	0.178	0.060	2.942	0.003	0.059	0.297
Factor 2	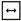	Factor 5	0.214	0.063	3.407	<0.001	0.091	0.337
Factor 2	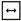	Factor 6	0.189	0.066	2.855	0.004	0.059	0.320
Factor 3	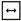	Factor 4	0.393	0.053	7.384	<0.001	0.289	0.497
Factor 3	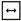	Factor 5	0.338	0.058	5.842	<0.001	0.225	0.451
Factor 3	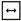	Factor 6	0.392	0.060	6.592	<0.001	0.276	0.509
Factor 4	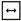	Factor 5	0.622	0.040	15.394	<0.001	0.543	0.701
Factor 4	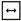	Factor 6	0.683	0.041	16.508	<0.001	0.602	0.764
Factor 5	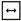	Factor 6	0.804	0.038	21.283	<0.001	0.730	0.878

**Table 8:** Scale Reliability Statistics.

	McDonald’s ω	Cronbach’s α
scale	0.856	0.837

Note. Of the observations, 429 were used, 0 were excluded listwise, and 429 were provided.

**Table 9:** Descriptive statistics.

		Mean	Standard Deviation	Variance	Rank	Minimum	Maximum
**Factor 1: Knowledge about eating and physical activity**							
Item 1	I know the benefits of practising physical activity every day.	4.43	0.788	0.622	4	1	5
Item 2	I differentiate the food groups of the food pyramid.	3.81	1.105	1.220	4	1	5
Item 3	I know the amount of each food group that we must consume every day according to the food pyramid.	3.38	1.136	1.290	4	1	5
Item 4	I differentiate foods rich in saturated fats (milk chocolate, packed chips, butter…) from foods rich in unsaturated fats (nuts, corn, avocado, sardines…).	3.89	1.140	1.299	4	1	5
**Factor 2: Habits about eating and physical activity**							
Item 5	I practice physical activity outdoors with my friends and relatives (siblings, cousins, parents…).	3.59	1.182	1.396	4	1	5
Item 6	I try to keep a healthy, varied and balanced diet.	3.67	1.099	1.207	4	1	5
Item 7	I avoid sweets.	3.00	1.239	1.535	4	1	5
Item 8	I search for information to improve my nutrition.	2.59	1.321	1.745	4	1	5
**Factor 3: Emotional health**							
Item 9	I identify my emotions and bear them in mind to feel good.	4.00	1.008	1.017	4	1	5
Item 10	I recognise my mood all the time.	4.00	1.071	1.147	4	1	5
Item 11	I establish priorities in my daily life and care less about those things that are not important.	3.61	1.124	1.264	4	1	5
Item 12	I love and accept myself as I am, with my flaws and virtues.	3.61	1.340	1.795	4	1	5
**Factor 4: Consumption of alcohol and drugs**							
Item 13	I know the consequences of consuming substances like cigarettes, shishas, alcohol or synthetic drugs.	4.67	0.775	0.601	4	1	5
Item 14	I know the diseases that derive from the consumption of cigarettes, alcohol and drugs.	4.58	0.792	0.628	4	1	5
Item 15	I am aware of the false ideas and hoaxes about tobacco, alcohol and drugs.	4.40	0.909	0.827	4	1	5
Item 16	I am aware that my friends can encourage me to consume alcohol, cigarettes or drugs.	4.27	1.097	1.204	4	1	5
**Factor 5: Social relationships and sexual activities**							
Item 17	I recognise the protection methods available to have safe sexual relations.	4.48	0.869	0.755	4	1	5
Item 18	I understand the importance of consent in sexual relations.	4.57	0.744	0.553	4	1	5
Item 19	I know sexually transmitted diseases and how to prevent them.	4.23	0.971	0.942	4	1	5
Item 20	I am aware of the gender differences that exist in society.	4.34	0.995	0.991	4	1	5
Item 21	I identify the attitudes that occur in a good couple relationship.	4.34	0.842	0.709	4	1	5
**Factor 6: Use of technologies**							
Item 22	I recognise the current risks in social networks (cyberbullying, sextorsion…).	4.55	0.697	0.486	4	1	5
Item 23	I make responsible use of my mobile devices (smartphone, tablet…).	4.13	0.985	0.971	4	1	5
Item 24	I know that, in the Internet, there is a lot of fake information about health.	4.47	0.856	0.732	4	1	5

## Data Availability

Not applicable.
